# Regenerative Potential of A Bovine ECM-Derived Hydrogel for Biomedical Applications

**DOI:** 10.3390/biom12091222

**Published:** 2022-09-02

**Authors:** Dalila Di Francesco, Fabio Bertani, Luca Fusaro, Nausicaa Clemente, Flavia Carton, Maria Talmon, Luigia Grazia Fresu, Francesca Boccafoschi

**Affiliations:** 1 Department of Health Sciences, University of Piemonte Orientale “A. Avogadro”, 28100 Novara, Italy; 2 Tissuegraft Srl, 15121 Alessandria, Italy

**Keywords:** tissue engineering, wound healing, angiogenesis, immunomodulation, decellularized extracellular matrix, natural hydrogel

## Abstract

Recent advancements in regenerative medicine have enhanced the development of biomaterials as multi-functional dressings, capable of accelerating wound healing and addressing the challenge of chronic wounds. Hydrogels obtained from decellularized tissues have a complex composition, comparable to the native extracellular environment, showing highly interesting characteristics for wound healing applications. In this study, a bovine pericardium decellularized extracellular matrix (dECM) hydrogel was characterized in terms of macromolecules content, and its immunomodulatory, angiogenic and wound healing potential has been evaluated. The polarization profile of human monocytes-derived macrophages seeded on dECM hydrogel was assessed by RT-qPCR. Angiogenic markers expression has been evaluated by Western blot and antibody array on cell lysates derived from endothelial cells cultured on dECM hydrogel, and a murine in vivo model of hindlimb ischemia was used to evaluate the angiogenic potential. Fibroblast migration was assessed by a transwell migration assay, and an in vivo murine wound healing model treated with dECM hydrogels was also used. The results showed a complex composition, of which the major component is collagen type I. The dECM hydrogel is biocompatible, able to drive M2 phenotype polarization, stimulate the expression of angiogenic markers in vitro, and prevent loss of functionality in hindlimb ischemia model. Furthermore, it drives fibroblast migration and shows ability to facilitate wound closure in vivo, demonstrating its great potential for regenerative applications.

## 1. Introduction

Tissue repair is a complex and highly regulated process needed for the correct healing and functional recovery of damaged tissues. In case of altered or ineffective healing processes, the result is a defective wound, which can lead to aesthetic problems, discomfort, a general chronic activation of inflammation and tissue function impairment [1]. This leads to a significant burden on both patients and the health system mainly because there is still a lack of knowledge of the molecular basis of the several processes involved in wound healing, resulting in a shortage of treatment options [2]. Thus, there is an increased need for therapies able to speed up this process in non-healing patients. In this context, tissue engineering is moving towards the development of strategies that can support tissue regeneration, such as the design and multi-functionalization of wound dressings with the ability to facilitate and accelerate the wound healing process by creating a regenerative environment [3,4].

To develop a multi-functional wound dressing, the many processes involved in wound healing should be considered [4]. In fact, wound healing involves different sequential and overlapping phases: a hemostatic/inflammatory phase, a proliferation phase and a remodeling phase [5]. During these steps, inflammation, coagulation, cell migration and proliferation, extracellular matrix (ECM) deposition, angiogenesis, cytokines, growth factors and enzymes, are all involved in assuring that healing proceeds correctly [6]. It is therefore important that factors such as inflammation, angiogenesis and cell migration are regulated during the healing process [7,8]; and this is what tissue engineering aims to achieve with the use of functionalized wound dressings. In this context, natural hydrogels represent exceptional candidates for regenerative applications [9,10].

Natural hydrogels are known for their biocompatibility, bioactivity, softness, and high-water content, which allows them to provide a moist environment able to soothe the injured area; moreover, their exceptional absorption capacity allows the removal of inflammatory exudate from direct contact with the wound [11,12]. Furthermore, one of the major advantages in using natural hydrogels for wound healing is represented by their composition, as they closely mimic the native ECM, offering an optimal environment for tissue repair and regeneration [11,13,14]. In fact, the native ECM presents a complex and dynamic network capable of providing structural support during tissue regeneration, as it supplies the necessary viscoelasticity thanks to its components, such as collagen, elastin, fibronectins, laminins, fibrin and hyaluronic acid (HA), and thus performs an essential role in wound healing [15]. During this process, in order to replace the original clot formation, ECM is synthesized by recruited fibroblasts and overlapped [7], thus influencing the re-epithelialization process and providing a provisional scaffold for cell adhesion, signaling the cells and influencing their behavior [8,16]. Moreover, ECM also plays a role during angiogenesis, as its structure and composition undergoes several changes to (i) allow the regulation of new blood vessels’ formation, (ii) modulate cell adhesion through integrins, and (iii) potentiate the angiogenesis [17,18].

As mentioned, angiogenesis is a necessary process for correct tissue repair, and its enhancement during the proliferative phase is important, as a high vascularization is required to meet all the metabolic needs [19]. However, an impaired angiogenesis leads to chronic wounds [20]; thus, the candidate scaffold for wound healing and regenerative application should be able to properly promote and sustain this process. Several hydrogels derived from natural sources can be used as basic elements of a proangiogenic system, and many have already demonstrated their potential in driving angiogenesis [21,22,23,24].

Finally, the management of inflammation during wound healing is also essential to avoid the chronic activation of inflammation and unpaired tissue repair; in this context, immunomodulation is a great resource [25]. Immunomodulation can be achieved through the macrophage polarization process, by switching the macrophages from the M1 phenotype, which represent the classic inflammatory phenotype, to M2 macrophages, the pro-regenerative phenotype [26]. M2 macrophages have a high phagocytic ability, they are involved in the production of ECM components, angiogenic and chemotactic factors, and can suppress the inflammatory response, providing the optimal environment for tissue repair [27,28]. The development of strategies able to drive macrophage polarization towards the M2 macrophages can be achieved by using naturally occurring biomaterials endowed with intrinsic anti-inflammatory signals. In many studies, natural biomaterials have proved to favor the regenerative M2 phenotype [29,30,31,32,33].

These findings prove that natural biomaterials show excellent potential for wound healing applications, mainly due to their ability to mimic the ECM’s physiological environment, by guiding essential processes such as angiogenesis and the immune response. Among natural biomaterials, those derived from decellularized ECM may demonstrate to be excellent candidates for this purpose [14]. Their biological advantage is mainly given by their complex structure and composition, which greatly conserves the native ECM, thereby maintaining the ability to support cellular behavior and tissue functions [34,35]. Moreover, these bioactive materials naturally possess both angiogenic and immunomodulatory potential [36,37,38,39]. This leads not only to a number of applications of decellularized ECM-derived materials in regenerative medicine [40], but also to the high interest in using them as multi-functional wound dressing [14,41,42].

The current study is aimed at evaluating the potential of a decellularized bovine pericardium extracellular matrix hydrogel for regenerative applications. The developed hydrogel has been characterized in terms of composition, ability to drive macrophage polarization towards the M2 regenerative phenotype, to promote angiogenesis and sustain endothelial cell function, and finally, to induce fibroblast migration sustaining the wound healing process.

## 2. Materials and Methods

### 2.1. Decellularization of the Bovine Pericardial Extracellular Matrix

Natural hydrogel obtained from the decellularized bovine pericardium extracellular matrix was kindly provided by Tissuegraft Srl (Alessandria, Italy) (Italian patent number 102020000007567, patented on 29 April 2022; International patent number PCT/IB2021/052779 submitted on 2 April 2021). The efficiency of the decellularization process, while maximally preserving the ECM proteins content, was evaluated by Tissuegraft and confirmed in this work. The lyophilized decellularized bovine pericardium extracellular matrix was then enzymatically digested to obtain a hydrolyzed extracellular matrix. The hydrolyzed extracellular matrix was used as such (from now on referred to as dECM) for matrix composition characterization and in vivo experiments. For all other in vitro experiments, it was used in the form of hydrogel (from now on referred to as dECM hydrogel), by allowing gelation of the dECM with a 30 min incubation at 37 °C.

### 2.2. Matrix Composition Characterization

#### 2.2.1. Silver Staining for Elastin and Collagen Content

To evaluate the presence of collagen and elastin in the dECM, a silver staining was used. The samples of either dECM, elastin standard (EPC, Elastin Products Company, Inc., Owensville, MO, USA) or type I collagen standard, which was extracted from rat tail tendons and solubilized in 0.02 N acetic acid for a final concentration of 4 mg/mL, finally sterilized and processed according to a previously reported protocol [43], were diluted in Laemmli Sample Buffer (Sigma Aldrich, Milan, Italy), and loaded in a 10% N,N′-Methylenebisacrylamide (acrylamide) (MerckMillipore 01709, Darmstadt, Germany) running gel for a Sodium Dodecyl Sulphate-PolyAcrylamide Gel Electrophoresis (SDS-PAGE). Silver staining was performed using the Pierce™ Silver Stain Kit (Thermo Fisher Scientific, Milan, Italy), according to the producer’s protocol.

#### 2.2.2. ELISA Kits for Glycosaminoglycan Content

To assess the presence of glycosaminoglycans (GAGs) and HA in the dECM, ELISA assay kits were used. In particular, the Glycosaminoglycans Assay Kit (Chondrex Inc., Woodinville, WA, USA) was used to determine the amount of GAGs. This kit permits dyeing of highly charged sulphated GAGs, excluding HA. The experiment was performed according to the producer’s protocol, using the assay protocol for samples containing extra proteins. The plate was read at 525 nm using the Victor X4 Multilabel Plate Reader (Perkin Elmer, Milan, Italy). The absorbance values were then processed using an Excel sheet (Version 2207; Microsoft Corporation, Redmont, WA, USA). The standard curve was plotted, and sample concentrations of GAGs calculated by a regression analysis.

To evaluate the concentration of HA in the dECM, the General Hyaluronic Acid ELISA kit (Byorbit, Milan, Italy) was used according to the producer’s protocol. The plate was read at 450 nm using the Victor X4 Multilabel Plate Reader. The absorbance values obtained were then processed using an Excel sheet (Version 2207; Microsoft Corporation, Redmont, WA, USA). The standard curve was plotted, and sample concentrations of HA were calculated by a regression analysis.

### 2.3. Cell Cultures

Human umbilical vein endothelial cells (HUVECs) (Sigma C-12203, Milan, Italy) were used to evaluate the expression of angiogenic markers. The cells were cultured in 100 × 20 mm cell culture dishes, maintained in Kaighn’s Modification of Ham’s F-12 Medium (F-12K) (ATCC 30-2004, Manassas, VA, USA), supplemented with 0.1 mg/mL heparin (Sigma Aldrich, Milan, Italy), 0.06 mg/mL endothelial cell growth supplement (ECGS) (Corning, Berlin, Germany), 1% penicillin, streptomycin, and amphotericin-B (PSF) (Euroclone, Nordhausen, Germany) and 10% fetal bovine serum (FBS) (Gibco, Milan, Italy). Cells were used up to the 8th passage.

NIH3T3-GFP (MyBioSource.com MBS168783, San Diego, CA, USA) fibroblast cells were used for transwell fibroblast migration assay. The cells were cultured in 75 cm^2^ flasks, maintained in Dulbecco’s Modified Eagle Medium (DMEM) (Gibco 21068-028, Milan, Italy), supplemented with 5 mM glutamine (Sigma Aldrich 1294808, Milan, Italy), 1% PSF and 10% FBS.

Normal human dermal fibroblast cells (NHDFs) (Lonza NHDF-Ad CC-2511, Basel, Switzerland) were used for transwell fibroblast migration assay. The cells were cultured in 75 cm^2^ flasks, maintained in Fibroblast Growth Medium- 2 BulletKit (FGM-2) (Lonza CC-3132, Basel, Switzerland). Cells were used up to the 8th passage.

Human monocytes were isolated by the Histopaque protocol (Sigma Aldrich, Histopaque^®^-1077, Milan, Italy) from anonymous human buffy coats provided by the Transfusion Service of the Ospedale Maggiore della Carità (Novara, Italy) after authorization from the local Ethics Committee (Comitato Etico Interaziendale Maggiore della Carità, Novara; authorization document 88/17). To study the differentiation of the monocytes towards different macrophage phenotypes, M0 cells were cultured for 6 days with RPMI 1640 (Lonza, Basel, Switzerland) with 20% FBS.

For experiments, cells were seeded on plate coated by the dECM hydrogel at a final concentration of 4 mg/mL. In all experiments, collagen hydrogels were used as control. The collagen hydrogels were prepared from the aforementioned type I collagen, by mixing the collagen solution with a buffer solution composed of DMEM, FBS, NaHCO_3_ 26 mM, NaOH 4 mM in PBS.

### 2.4. RNA Isolation and Real-Time Polymerase Chain Reaction

RT-qPCR was used to access the expression of M1 and M2 macrophage phenotype markers of human monocytes left to differentiate into M0 on 4 mg/mL dECM hydrogel coatings. 250,000 human monocytes were seeded in each well as before mentioned. RNA was extracted by TRIzol (Thermo Fisher Scientific, Milan, Italy). Retro-transcription was performed using the High-Capacity RNA-to-cDNA™ Kit (Applied Biosystems, Waltham, MA, USA), according to the manufacturer’s instructions. A two-step cycling real-time PCR was carried out in a volume of 10 μL per well in a 96-well optical reaction plate (Biorad, Hercules, CA, USA). PCR mix was prepared according to SsoAdvanced Universal SYBR Green Supermix (BioRad, Hercules, CA, USA) protocol with forward and reverse primer 400 nM and 1 μL of cDNA template. Glyceraldehyde 3-phosphate dehydrogenase (GADPH) was used for normalization. The primers are summarized in Table 1. The relative quantification was determined by the ΔCT method.

### 2.5. Cell Lysates

For in vitro angiogenic potential evaluation, HUVEC cell lysates were prepared, in order to perform Western blot analysis of endothelial nitric oxide synthase (eNOS) and Human Angiogenesis Antibody Array kit for 20 pro and anti-angiogenic marker expression. 4 mg/mL dECM hydrogel coatings were prepared in a 24 well plate, 200 µL hydrogel coatings were made, and the plate was then kept at 4 °C for 1 h, to avoid gelation. The coatings were then removed and 100,000 HUVECs/well were seeded and maintained in F-12K 10% FBS. Cell lysis was performed on day 1 and 3. 24 h before cell lysis, cells were starved by using the maintenance media with F-12K without FBS (referred as starved control). For the Western blot samples, enrichment with vascular endothelial growth factor (VEGF) was also tested: 50 ng/mL VEGF (Sigma Aldrich, V7259, Milan, Italy) was added to the media of control, dECM hydrogel and starved control.

Chemical and mechanical cell lysis were performed. Protein quantification of cell lysates was then performed with Pierce BCA Protein Assay Kit (Thermo Fisher, Milan, Italy) according to the producer’s protocol.

### 2.6. Western Blot

Western blot was used to assess the expression of angiogenic marker eNOS, and the expression of tubulin for normalization. Lysate samples were prepared as previously described and 50 µg of protein loaded in a 10% N,N′-Methylenebisacrylamide (acrylamide) (MerckMillipore 01709, Darmstadt, Germany) running gel. After SDS-PAGE electrophoresis, Western blot transfer was performed. The nitrocellulose membranes were incubated overnight with rabbit anti-eNOS, 130 kDa (MerckMillipore, SAB4502014, Darmstadt, Germany) and mouse anti-tubulin, 50 kDa (MerckMillipore, 05-829, Darmstadt, Germany) primary antibodies, followed by 1 h incubation with the secondary anti-rabbit and anti-mouse antibodies conjugated with Horse Radish Peroxidase (HPR) (Perkin Elmer, Milan, Italy). The substrate used for chemiluminescence detection was the Western Lightning Plus Enhanced Chemiluminescence Substrate kit (ECL) (Perkin Elmer, Milan, Italy). The membranes were then analyzed at ChemiDoc (Bio-Rad, Hercules, CA, USA) for chemiluminescence detection. For densitometry “Image Lab” (Version 6.0.1; Bio-Rad, Hercules, CA, USA) software was used.

### 2.7. Human Angiogenesis Antibody Array

Human Angiogenesis Antibody Array (Abcam ab134000, Milan, Italy) is an antibody-pair-based assay. Capture antibodies are supplied arrayed/spotted on a membrane, with each pair of spots representing a different analyte, and are as follows: Angiogenin, Epidermal Growth Factor (EGF), C-X-C motif chemokine 5 (ENA-78), basic Fibroblast Growth Factor (bFGF), Growth-Regulated Oncogene (GRO), Interferon-gamma (IFN-γ), Insulin-like Growth Factor-1 (IGF-I), IL-6, IL-8, Leptin, Monocyte Chemoattractant Protein-1 (MCP-1), Platelet Derived Growth Factor (PDGF-BB), Placental Growth Factor (PlGF), Chemokine (C-C motif) ligand 5 (RANTES), Transforming Growth Factor-beta (TGF-beta1), Tissue inhibitors of metalloproteinases 1 and 2 (TIMP-1 and TIMP-2), Thrombopoietin, VEGF-A and VEGF-D. The order of the membrane’s spots is shown in Table 2.

150 µg/mL of the cell lysis sample were used according to the producer’s protocol. The membranes were then analyzed at ChemiDoc (Bio-Rad, Hercules, CA, USA) for chemiluminescence detection. The comparison has been performed using ImageJ for a semi-quantitative analysis.

### 2.8. Hind Limb Ischemia Model

The in vivo experiments have been authorized by Ministero della Salute (Authorization n° 779/2021-PR Risp. a prot. DB064.73). The mice were bred under pathogen-free conditions in the animal facility of the University of Piemonte Orientale, Department of Health Sciences (Authorization n° 217/2020-PR) and treated in accordance with the Ethical Committee and European guidelines.

The hind limb ischemia in vivo model has been widely used to assess the angiogenic potential of biomaterials, using a protocol adapted from Brenes et al. [44], and Ungerleider et al. work [45]. First of all, BALB/c male mice were anesthetized by an intraperitoneal injection of Zoletil^®^ + Xylazine (47.5 mg/kg and 16 mg/kg, respectively). To prevent the negative effects of anesthesia, the animals were placed on a heated pad and a humectant eye gel was applied for the entire time of the surgery. The femoral artery was isolated from other blood vessels, tendons, and annexes with the help of a stereomicroscope; it was then cauterized with the aid of a thermal cautery unit. On the cauterized femoral artery, 50 µL of either PBS, 4 mg/mL collagen or 8 mg/mL dECM was placed. Moreover, intramuscular injections of samples were also performed in the gracilis muscle of the cauterized limb, consisting in 50 µL of sample in 4 distinct points. Afterwards, the skin was stitched with adsorbable suture 5.0 and the mice were kept under a red hot lamp until full awakening. Mice were checked daily up to 28 days to assess their ability to use the limb and the possible presence of necrosis. A functional score was given, based on visual evaluation, according to the Tarlov scale, ischemia scale, and modified ischemia scale [44]. At day 15 and 28, mice were sacrificed to obtain a limb specimen for histological evaluation. Histological sample preparation was performed after 4% formalin fixation for a day. Samples were then dehydrated, cleared and embedded in paraffin, and 5 µm thick sections were obtained. The sections were then deparaffinized, rehydrated, and stained with hematoxylin-eosin (H&E stain) for light microscopy observation. The experiment was performed in triplicate for all the tested conditions.

### 2.9. Transwell Migration Assay

Corning^®^ Transwell^®^ polyester membrane inserts (0.4 μm pore, Sigma Aldrich CLS3470, Milan, Italy) were placed in 24 well plates. 4 mg/mL dECM hydrogels were prepared by diluting the dECM with cell media; 100 µL of hydrogel was placed onto the upper chamber of the transwells and the well plate was then maintained at 37 °C for 30 min to allow the gelation of the hydrogel. Once the dECM hydrogel was gelled, NHDF and NIH3T3-GFP cells were seeded on top of the dECM hydrogel at a cell concentration of 5000 cells per well; cells were also seeded directly onto transwells’ membranes as control. To enhance cell migration, a higher FBS medium concentration was added to the lower chamber. In particular FGM-2 10% FBS for NHDF cells and DMEM 10% FBS for NIH3T3-GFP cells was placed in the lower chamber, while 2% FBS concentrations were used on the upper membrane.

At day 1, 3 and 7 the transwells and hydrogels were fixed with 4% formalin for 1 h. The samples were then rinsed with PBS and cell migration was evaluated.

For NIH3T3-GFP cells, both the lower part of the transwell membrane and the hydrogel were observed with fluorescence microscopy.

For NHDF cells, the fixed transwells were stained with a 0.5% crystal violet solution (Sigma Aldrich, Milan, Italy) for 30 min. Following staining, the transwells were rinsed with deionized water until no colour was present. The transwells were then observed with optical microscopy.

### 2.10. Wound Healing In Vivo Model

BALB-c male mice were bred under pathogen-free conditions in the animal facility of the University of Piemonte Orientale, Department of Health Sciences (Authorization n° 217/2020-PR) and treated in accordance with the Ethical Committee and European guidelines. Before the induction of wounds, mice were anesthetized with 3% isoflurane and their back was shaved. The wounds were generated on the back using a 4 mm puncher (Kai Medical, Solingen, Germany). They were then treated with PBS as control, 4 mg/mL collagen or 8 mg/mL dECM. 30 µL of sample was applied on the wounds. The wounds were measured with a metric caliber daily for up to 7 days to evaluate the closure of the wound, with addition of the PBS solution, collagen or dECM on the wounds every 2–3 days. After wound closure, representative specimens from mice of each condition were isolated, to evaluate the histological structure and inflammatory infiltration. The specimens were fixed in 4% formalin for a day, then dehydrated and cleared, and finally embedded in paraffin. Paraffin embedded samples were cut into thin 5 µm slices, transversally to the wound, and hematoxylin eosin staining was performed for subsequent optical microscopy evaluation.

### 2.11. Statistical Analysis

Sample media and standard deviation of the RT-qPCR, Western blot, Human Angiogenesis Antibody Array kit and wound healing in vivo model experimental results were calculated. On this data, a one tail Student’s T statistical test for homoscedastic samples was performed on the software “Quantitative Skills SISA” to verify the statistical significance (significance level of 5%). The differences between variables with a value of *p* < 0.05 were considered statistically significant.

## 3. Results

### 3.1. Matrix Composition Characterization

Characterization of the matrix macromolecular content was performed to evaluate the presence of collagen, elastin, GAGs, and HA in the dECM after decellularization and following processing.

**Figure 1:**
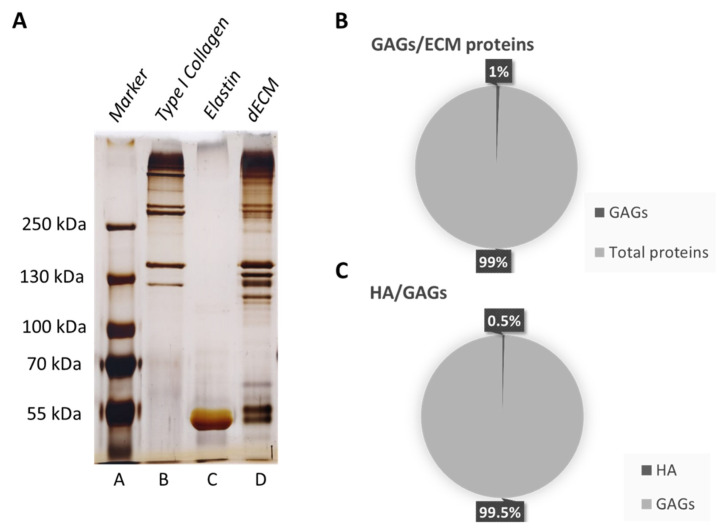
Matrix composition characterization. (**A**) Silver staining on SDS-PAGE gel electrophoresis. A: Protein marker peqGold, B: collagen type I standard, C: elastin standard and D: dECM. (**B**) ELISA evaluation of GAGs in dECM. Percentage presence of GAGs over the total dECM proteins. (**C**) ELISA evaluation of HA in dECM. Percentage presence of HA over the total GAGs amount.

A Figure 1A shows the silver staining performed on the SDS-PAGE gel. dECM (lane D) is mostly made up of type I collagen, as the standard bands correspond in the dECM lane; moreover, the presence of elastin can also be appreciated, corresponding at ~60 kDa.

The presence of other bands not corresponding to any of the used standards shows that other proteins are present, whose molecular weight might correspond to GAGs, which range in molecular weight from 6 to 100 kDa [46]. Based on this, specific ELISA tests have been performed, demonstrating that 1% of the dECM is made up of sulphated GAGs and 0.5% of HA (Figure 1B,C).

### 3.2. Immunomodulatory Potential

**Figure 2:**
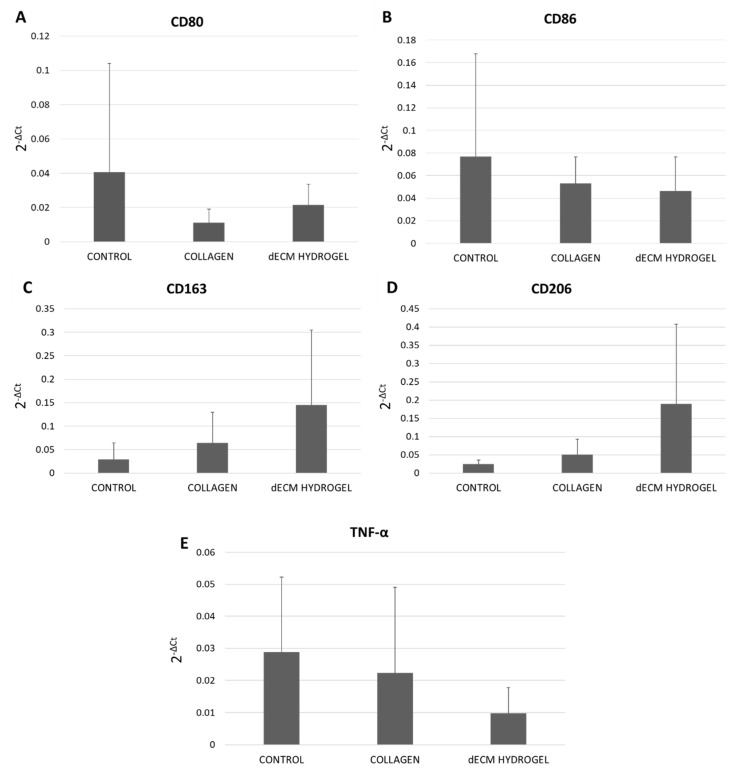
Immunomodulatory potential of dECM hydrogel. (**A**) Gene expression of M1 phenotype marker CD80 in monocyte-derived macrophages on 4 mg/mL dECM hydrogel coatings. (**B**) Gene expression of M1 phenotype marker CD86 in monocyte-derived macrophages on 4 mg/mL dECM hydrogel coatings. (**C**) Gene expression of M2 phenotype marker CD163 in monocyte-derived macrophages on 4 mg/mL dECM hydrogel coatings. (**D**) Gene expression of M2 phenotype marker CD206 in monocyte-derived macrophages on 4 mg/mL dECM hydrogel coatings. (**E**) Gene expression of TNF-α in monocyte-derived macrophages cultured on 4 mg/mL dECM hydrogel coatings. Results are expressed as 2^−ΔCt^. Data are means ± S.D. of 6 independent experiments from distinct donors.

Human monocytes were cultured on 4 mg/mL dECM hydrogel coatings for 6 days. RT-qPCR was used to assess the expression of M1 phenotype markers CD80 and CD86, as shown in Figure 2A,B, and of M2 phenotype markers CD163 and CD206, as displayed in Figure 2C,D. TNF-α expression was also evaluated to assess the presence of inflammation (Figure 2E). Our results demonstrate that the dECM hydrogels show a tendency to reduce the expression of pro-inflammatory marker CD80/CD86 (Figure 2A,B), while increasing the expression of anti-inflammatory CD163 and CD206 (Figure 2C,D). Moreover, as shown in Figure 2E, the expression of TNF-α is lower in dECM hydrogels compared to the collagen hydrogel and the control. Although not significant, these results show the dECM’s tendency to skew macrophages M0 towards the M2 phenotype.

### 3.3. Angiogenic Potential

#### 3.3.1. Western Blot

**Figure 3:**
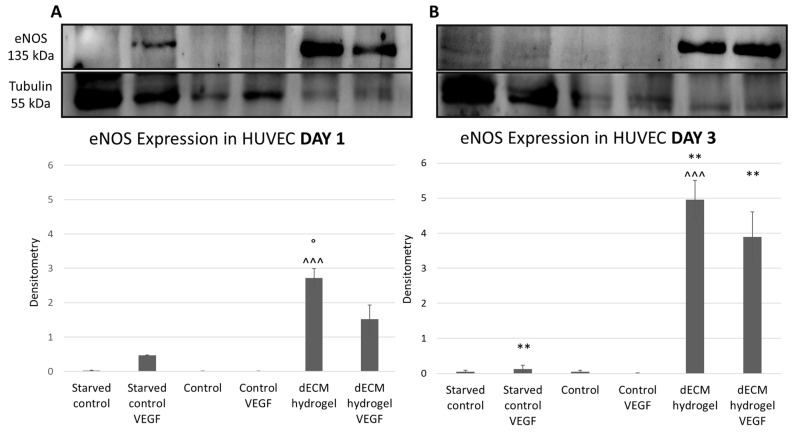
Western blot for eNOS expression. HUVECs were cultured on 4 mg/mL dECM hydrogel coatings, with and without 50 ng/mL of VEGF. (**A**) eNOS normalized expression at day 1. (**B**) eNOS normalized expression at day 3. Tubulin expression was used to normalize results. Images are representative of the experiment which has been repeated in triplicate. Data are means ± S.D. of 2 experiments. ^^^: *p* < 0.001 dECM hydrogel vs control; **: *p* < 0.01 day 3 vs day 1; °: *p* < 0.05 dECM hydrogel vs dECM hydrogel VEGF.

The expression of angiogenic marker eNOS in cell lysates of HUVECs cultured onto 4 mg/mL dECM hydrogel coatings for 1 (Figure 3A) and 3 (Figure 3B) days was evaluated by Western blot. The results were normalized using tubulin’s expression. The results show that dECM hydrogels induce the expression of eNOS, compared to the controls, in a time dependent manner, with the highest effect after 3 days (Figure 3B), as the expression is statistically significantly increased at day 3 in the dECM hydrogel condition. Moreover, there is an important increase in eNOS expression by HUVECs cultured on the dECM hydrogel coatings compared to control, as only a moderate expression is present in the positive control represented by HUVECs starved and enriched with VEGF, which is then lost by day 3, as VEGF stimulation was not renewed during the experiment.

#### 3.3.2. Human Angiogenesis Antibody Array

**Figure 4:**
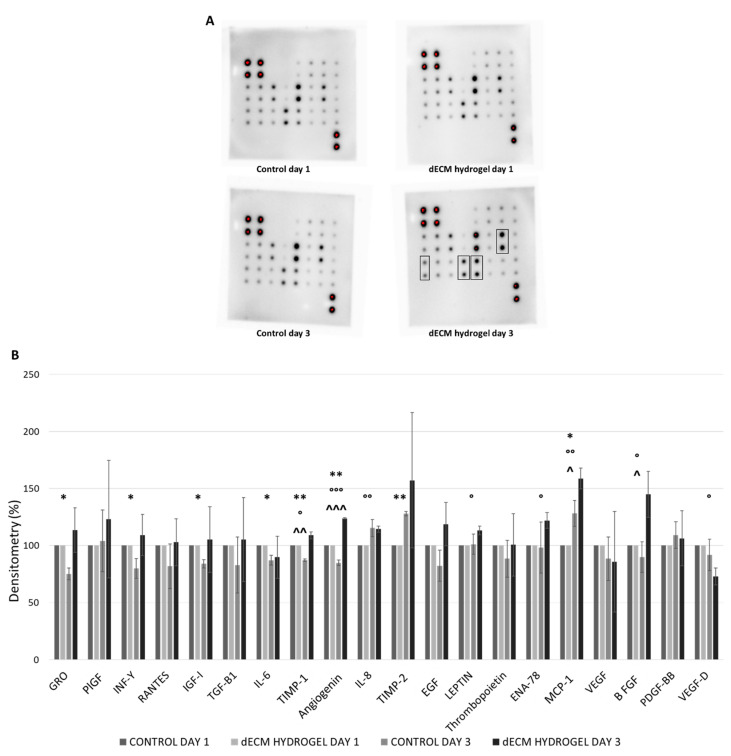
Angiogenic potential of dECM hydrogel. (**A**) Human Angiogenesis Antibody Array on 4 mg/mL dECM hydrogels: spot arrays in controls and hydrogels at days 1 and 3. Thicker spots on dECM hydrogel day 3 are marked in boxes. (**B**) Human Angiogenesis Antibody Array relative percentage expression of markers by HUVECs in controls and 4 mg/mL dECM hydrogels at days 1 and 3. Data are normalized according to producer’s protocol and are means ± S.D. of 3 experiments. ^: dECM hydrogel vs control at day 3; *: control day 3 vs control day 1; °: dECM hydrogel day 3 vs dECM hydrogel day 1. *, °, ^: *p* < 0.05; **, °°, ^^: *p* < 0.01; °°°, ^^^: *p* < 0.001.

This angiogenesis antibody array kit was used to assess the expression of different pro- and anti-angiogenic markers in cell lysates of HUVECs cultured on 4 mg/mL dECM hydrogel coatings for 1 and 3 days. Figure 4A shows the spot array of controls and hydrogels at days 1 and 3, while in Figure 4B the normalization of results is reported as a relative percentage, calculated considering the positive controls and day 1 control as the reference array. As shown in Figure 4A, the increased expression of some angiogenic factors can be appreciated (thicker array spots marked in boxes), such as PlGF, which is a member of the VEGF family, MCP-1, a major regulator of monocyte/macrophage migration, and TIMP-1 and TIMP-2, from HUVECs cultured on 4 mg/mL dECM hydrogels for 3 days.

In particular, a statistically significant increase of expression of TIMP-1, Angiogenin, IL-8, LEPTIN, ENA-78, MCP-1 and bFGF is noted in HUVECs cultured on dECM hydrogel for 3 days, when compared to control (Figure 4B). Meanwhile, the expression of several angiogenic markers by the control condition is lost at day 3. Hence, the results demonstrate a general trend of angiogenic factors increase in HUVECs cultured on dECM hydrogel coatings for 3 days compared to control. Hence, the project moved towards the evaluation of this potential with in vivo studies.

#### 3.3.3. Hind Limb Ischemia In Vivo Model

Hind limbs functionality and morphology of mice, whose femoral artery was cauterized and treated with PBS, 4 mg/mL collagen or 8 mg/mL dECM, was assessed for 28 days. By visual evaluation, the mice were given a functional score, depending on the limb functionality, the presence of gangrenous tissue and necrosis. Table 3 summarizes the score for each mouse, according to the Tarlov, Ischemia and Modified Ischemia scores [44]. The table shows the scores given at 2, 7, 14, 21 and 28 days after treatment, to verify the functionality and morphological aspect of the limb upon time. The general trend was the loss of limb functionality and development of gangrenous tissue for PBS-treated mice, with no amelioration during the 28 days course (as seen in the Video S1, PBS treated HLI model), and instead a functional recovery for collagen and dECM-treated mice, which were able to regain full, weight bearing walking in the 28 days course (as seen in Video S2, collagen treated HLI model, and Video S3, dECM treated HLI model).

**Figure 5:**
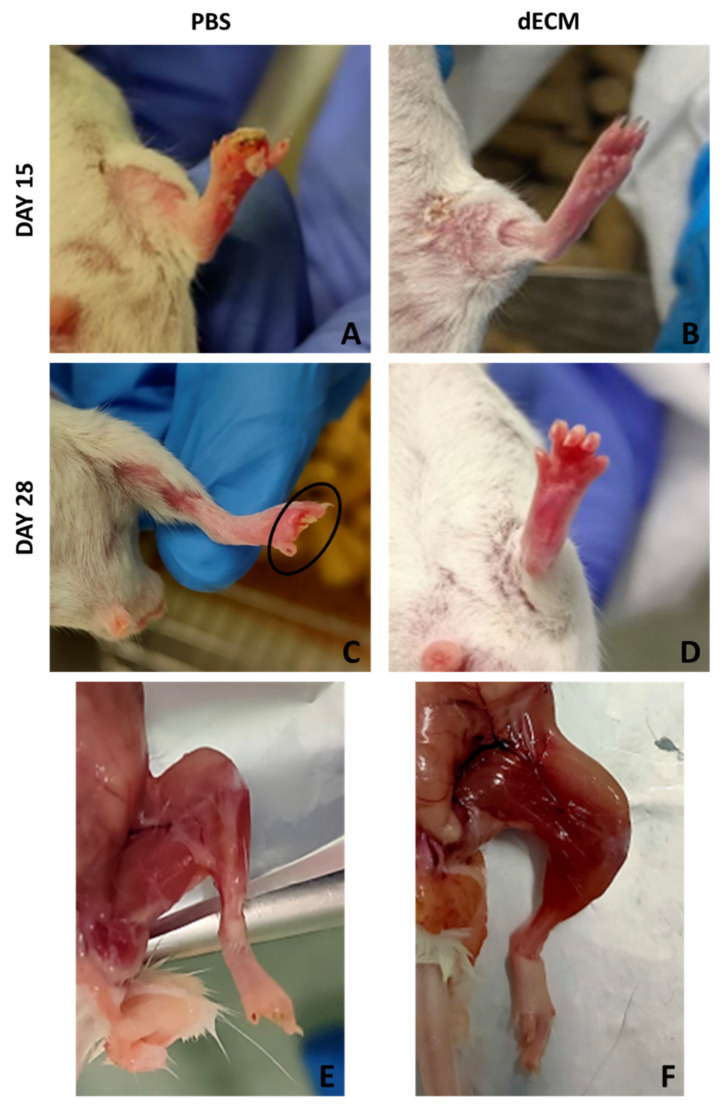
Hind limb ischemia model macroscopic evaluation. (**A**,**B**) hind limbs of BALB/c mice treated with PBS and dECM at day 15. (**C**,**D**) hind limbs of BALB/c mice treated with PBS and dECM at day 28. (**E**,**F**) hind limbs of BALB/c mice treated with PBS and dECM at day 28, after sacrifice.

Figure 5 shows macroscopic evaluation of the mice 15 and 28 days after treatment with PBS and dECM. Mice treated with collagen and 8 mg/mL dECM regained the limbs’ functionality very fast and showed no signs of gangrenous tissue or necrosis (Figure 5B,D); on the other hand, mice treated with PBS lost functionality of the limb and showed necrosis, with the loss of one or more toes (Figure 5A,C). Moreover, upon sample collection, PBS-treated mice displayed a slight level of atrophy due to the loss of functionality of the limb (Figure 5E), while this was not present in dECM-treated mice (Figure 5F).

**Figure 6:**
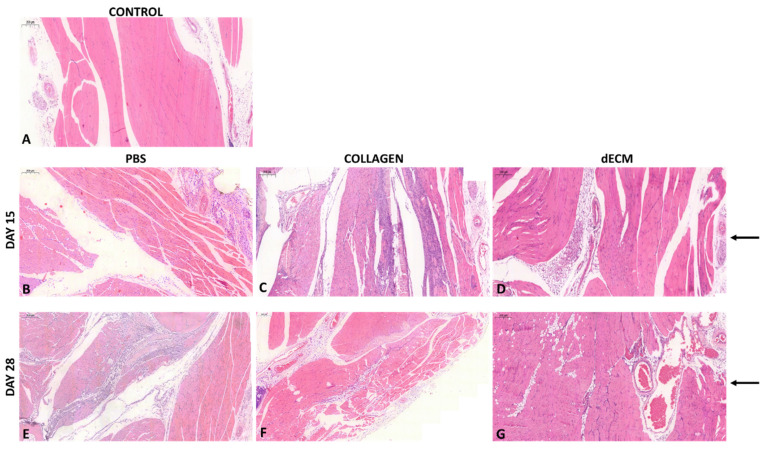
Histological samples of BALB/c mice. (**A**) native structure of a male BALB/c mouse, (control). (**B**–**D**) hind limbs isolated from mice treated with PBS, collagen and 8 mg/mL dECM at day 15. (**E**–**G**) hind limbs isolated from mice treated with PBS, collagen and 8 mg/mL dECM at day 28. The arrows highlight the injection site and the presence of vessels. 5× magnification. Scale bar 200 μm.

On day 15 and 28, mice were sacrificed for specimen collection for the histological analysis. Figure 6A shows a non-cauterized mouse’s histological specimen of the limb, used as control (to show how the native tissue of limb appears). As shown in Figure 6, PBS alone and collagen induced a significant inflammatory infiltration in the mouse limb, both at 15 (Figure 6B,C) and 28 (Figure 6E,F) days. Interestingly, the 8 mg/mL dECM-treated limb did not show any alteration in the histological architecture either after 15 (Figure 6D) or 28 (Figure 6G) days. Arrows highlight the presence of vessels formed at the injection site, where a substantial neo angiogenesis is present in mice treated with dECM.

### 3.4. Wound Healing Potential

#### 3.4.1. Transwell Migration Assay

**Figure 7:**
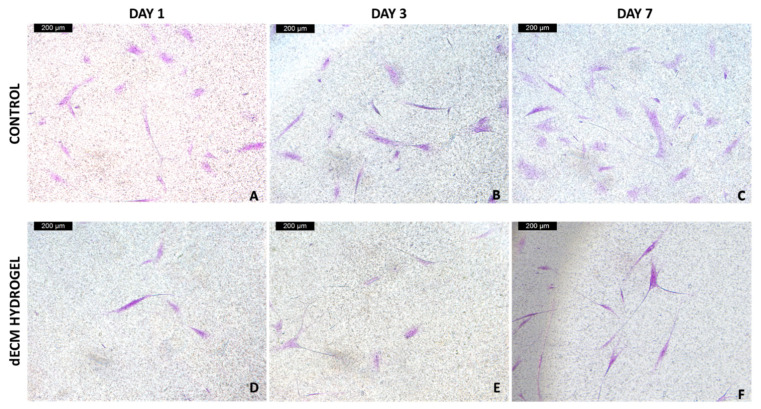
Crystal violet staining of the migrated NHDF. (**A**,**D**) migrated NHDF at the bottom of control and dECM hydrogel transwells at day 1. (**B**,**E**) migrated NHDF at the bottom of control and dECM hydrogel transwells at day 3. (**C**,**F**) migrated NHDF at the bottom of control and dECM hydrogel transwells at day 7. Magnification 10×. Scale bar 200 μm.

NIH-GFP and NHDF fibroblasts were cultured for 7 days on 4 mg/mL dECM hydrogels, and placed on transwells, to assess the ability of the hydrogel to drive migration and wound closure potential. NHDF cells samples were fixed at days 1, 3 and 7, and the presence of migrated fibroblasts on the bottom of the transwell was evaluated using crystal violet staining. Figure 7 shows the acquired images of the bottom of control and dECM hydrogel transwells, at 1, 3 and 7 days, where we can appreciate an increasing presence of migrated fibroblasts in the hydrogel condition.

**Figure 8:**
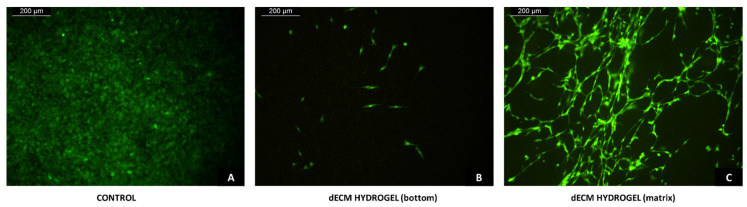
Transwell fibroblast migration. (**A**,**B**) NIH-GFP at the bottom of control and dECM hydrogel transwells after 7 days from seeding. (**C**) Migrated NIH-GFP inside the 4 mg/mL dECM hydrogel after 7 days from seeding. Magnification 10×. Scale bar 200 μm.

NIH3T3-GFP fibroblasts samples were fixed at day 7, then the presence of migrated fibroblasts inside the dECM hydrogel and on the bottom of the transwell was evaluated with fluorescence microscopy. Figure 8A,B shows the acquired images of the bottom of control and dECM hydrogel transwells, respectively, where we can appreciate the presence of migrated fibroblasts in both conditions, even if in the presence of the hydrogel there is a lower number of migrated cells. Figure 8C shows the matrix inside of the fixed 4 mg/mL dECM hydrogel, where a network of migrated fibroblasts has formed inside the hydrogel.

#### 3.4.2. Wound Healing In Vivo Model

**Figure 9:**
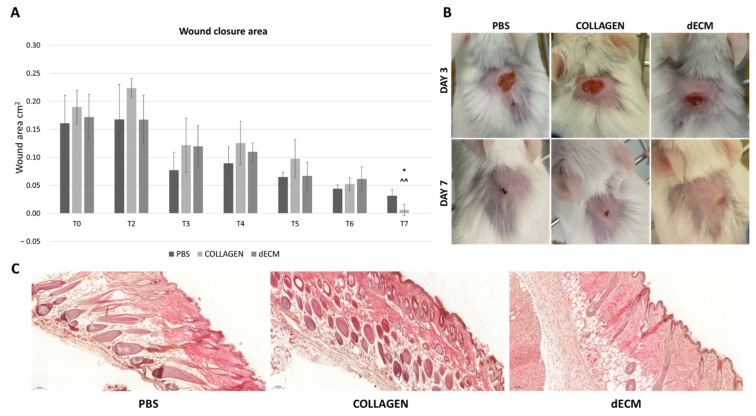
Wound healing model. (**A**) Wound area, expressed in mm^2^, of wound treated with PBS, collagen and 8 mg/mL dECM, from day 1 to day 7. Data are means ± S.D. of 3 samples for each condition. *: *p* < 0.05 collagen vs control; ^^: *p* < 0.01 dECM vs control. (**B**) representative images of wound area in wound healing models treated with PBS, collagen and dECM at day 3 and 7. (**C**) histological samples of transverse wound areas. The images display the tissue architecture of wounds treated with PBS, collagen and 8 mg/mL dECM at day 7. Magnification 10×.

Wounds (Ø 4 mm) were generated on the back of BALB/c mice, and then treated with PBS, 4 mg/mL collagen or 8 mg/mL dECM. The wounds were measured daily up to 7 days, to assess the healing potential of the dECM. As shown in Figure 9A, the area of wound treated with the 8 mg/mL dECM is completely closed on day 7, while wounds treated with PBS and collagen hydrogels are not fully healed. Figure 9B shows representative images of the PBS, collagen and dECM treated mice at day 3 and 7.

On day 7, specimens from each condition were collected for histological analysis. Figure 9C shows the histological samples, prepared by cutting transversally through the wound, to assess the presence of structural alterations and inflammation in the area. The results reveal no tissue alteration in any of the conditions, with the presence of hair regrowing at wound area and of new blood vessels.

## 4. Discussion

The development of a bioactive and natural biomaterial starting from animal derived extracellular matrix is currently of great interest, but still represents a challenge. In fact, the decellularization process must aim at removing the cellular component while preserving the composition of extracellular matrix, as its complexity and richness represents one of the main advantages of using dECM as a biomaterial for regenerative purposes [47]. Our results suggest that the standardized process used to obtain the decellularization and solubilization of the dECM allows to preserve the main components of the extracellular matrix (i.e., collagen, elastin, and GAGs), while efficiently removing the cellular component. The complex composition of this matrix provides a biological benefit, closely mimicking the physiological environment and, therefore, sustaining cellular and tissue functionality, and maintaining the ability to guide tissue regeneration [34,48].

The management of the inflammatory response and the induction of a regenerative environment can be achieved with immunomodulation, and, in particular, with the ability to drive macrophage polarization [27]. Thus, it was noteworthy to evaluate the effect of the dECM hydrogel on monocyte differentiation towards M1 or M2 macrophages. Although not significant, due to the intrinsic variability of the cells used, obtained from distinct human donors, our results support an immunomodulatory role of the dECM with a tendency to skew macrophages M0 towards the M2 phenotype. Moreover, the dECM hydrogel is also able to maintain the expression of M1 phenotype markers and of pro-inflammatory cytokine TNF-α below the control expression. These data demonstrate not only the excellent biocompatibility of the dECM hydrogel, but also its ability to provide regenerative cues.

Angiogenesis plays a key role in adequate tissue regeneration and, therefore, the expression of angiogenic markers was evaluated. The expression of eNOS, by endothelial cells cultured on dECM hydrogels was first assessed, as it is well known that eNOS is the major nitric oxide synthase isoform involved in angiogenesis, therefore increasing nitric oxide (NO) production under VEGF stimulation [49]. eNOS is also the mediator of VEGF-induced vascular permeability, a process necessary during wound healing [50]. Thus, eNOS expression by endothelial cells is essential for angiogenesis and wound healing. The results show that eNOS expression is only moderate in the positive control, represented by HUVECs starved and enriched with VEGF. Instead, the dECM hydrogel not only sustains, but also induces an important time-dependent increase in eNOS expression when compared to control. Interestingly, the results reveal how the dECM hydrogel alone is able to significantly drive this effect, regardless of adding VEGF, which therefore does not represent an advantage. With the use of a cytokine array, it is also demonstrated that other angiogenic markers have a significant time-dependent increase in expression when HUVECs are cultured on dECM hydrogels. Specifically, TIMP-1, Angiogenin, IL-8, LEPTIN, ENA-78, MCP-1 and bFGF are increased at day 3. bFGF is known to stimulate the proliferation of the endothelial cells, to promote macrophage and fibroblast migration to the damaged tissue to heal epidermal wounds [51], while angiogenin is a secreted multi-functional ribonuclease involved in cell proliferation, survival, migration and differentiation. Angiogenin is also important in wound neovascularization: in fact, when endothelial cells are subjected to damage, high concentrations of this ribonuclease may facilitate rapid blood vessel formation and tissue repair, suggesting that angiogenin may substantially promote wound healing [52]. In our experiments, angiogenin is increased at day 3 on dECM hydrogels, while its expression is significantly decreased at day 3 by the control. MCP-1 is an angiogenic chemokine responsible for the migration and activation of monocytes and macrophages. Moreover, it upregulates many genes involved in angiogenesis, like the angiogenic factors previously mentioned, but also adhesion molecules, matrix proteins, proteases and their inhibitors [53]. IL-8 is a well-known angiogenic factor: it is able to enhance endothelial cell growth and survival factors, and it plays a role in endothelial cell and neutrophil migration [54,55]. LEPTIN is also an angiogenic factor which shows the ability to promote endothelial proliferation and differentiation in vitro, inducing tissue regeneration in vivo [56,57]. Due to the presence of a particular ELR motif, ENA-78 possesses strong angiogenic activity; in fact, it is currently being investigated as a predictive factor for wound healing, showing promising results [58,59]. Lastly, the results show an upregulation of tissue inhibitors of metalloproteinases (TIMP-1), in particular, by day 3, TIMP-1 is significantly increased on dECM hydrogels but significantly reduced in the control. TIMPs are regulators of ECM turnover, tissue remodeling, and cellular behavior; thus, they have a role in the modulation of angiogenesis, cell proliferation, and apoptosis [60]. They are generally anti-angiogenic markers, therefore their higher expression contrasts with the other results. Theoretically, these high concentrations could be explained by two reasons: first is the stimulation of TIMPs is due to elevated levels of MCP-1 expression [53]. Second, TIMPs’ activation must be correlated to a high activation of metalloproteases (MMPs), particularly for angiogenesis, as TIMPs play an important role in the stabilization of capillary networks during this process [61]. Moreover, higher levels of MMPs and MMP/TIMPs ratio demonstrate a role in non-healing wounds [62]. Thus, a higher concentration of TIMPs may be considered helpful for our goals; however, this aspect needs to be further investigated. All together, these results proved the angiogenic potential of the dECM, so an in vivo evaluation was performed, and very encouraging results were achieved. Mice treated with PBS show a rapid development of necrosis and loss of limb’s functionality; while mice treated with 8 mg/mL dECM quickly regain their limb’s functionality and do not show any macroscopical alterations. When looking at the histology, mice treated with PBS and collagen reveal a certain inflammatory infiltrate, both at day 15 and 28 after treatment, even though there are no significant alterations in the microstructure of the tissue. However, in accordance with the previous in vitro results, no grade of inflammatory infiltrate is seen in mice treated with the dECM, proving once again its excellent biocompatibility, and supporting the regenerative process. Furthermore, the tissue is highly vascularized in the dECM injection area, where the femoral artery was cauterized, contributing to the rapid recovery of the mice’s limb.

As fibroblast migration is pivotal for wound healing, the dECM hydrogel’s ability to drive fibroblast migration was evaluated. The results, obtained with two different cell lines of fibroblasts, demonstrate that fibroblasts in presence of dECM hydrogels are able to migrate through the matrix and through the transwell membrane. As expected, more fibroblasts are found at the bottom of control transwells, because fibroblasts seeded on the dECM hydrogels need more time to reach transwell membranes, as they first have to migrate through the hydrogel itself. Furthermore, we also found that the dECM hydrogels are able to provide a suitable microenvironment for the fibroblasts to form a cellular network in a three-dimensional environment. These results prove once more that the rich environment of ECM interacts closely with cells, driving their migration and significantly contributing to tissue regeneration [63].

Finally, the results achieved led to in vivo validation with a wound healing model. The results confirm that in 7 days, the dECM was able to completely heal the wounds, repairing the damaged area significantly faster than wounds treated with either PBS or collagen hydrogel. Histological results also show that through the area once injured, an unaltered and highly vascularized tissue formed, with no presence of inflammatory infiltrate. This further supports the potential of dECM as a bioactive and multi-functional wound dressing.

Ultimately, the findings of the present study complement the current research on animal dECM-derived biomaterials for wound healing and regenerative medicine applications, further proving the importance of exploiting ECM components to achieve bioactivity. Moreover, there are other advantages to using animal derived biomaterials, such as: (i) the low immunogenicity of the product, guaranteed by the decellularization process used, should reduce the risk of rejection after implant [36,41] and, (ii) being a large animal derived food industry by-product, it should be available in large quantities and easily obtainable when compared to cell derived or human sources of dECM [35]. However, there are still some disadvantages and risks in using these biomaterials. Among these, the lack of standardized decellularization and sterilization techniques remains a concern, due to the risk of viral, microbial, and chemical contaminations of the product [64]. Furthermore, batch-to-batch variation and absence of manufacturing considerations also represents disadvantages [65]. These limitations lead to the necessity for further preclinical investigations of animal derived-dECM biomaterials before their clinical and industrial translation can be achieved.

## 5. Conclusions

The aim of this work was to evaluate the regenerative ability of a dECM hydrogel derived from bovine pericardium, along with the characterization of its immunomodulatory and angiogenic abilities. The results obtained proved the extraordinary potential of the dECM for regenerative applications, as it not only has immunomodulatory effect promoting the functional regeneration of the tissue, but it is also able to provide a regenerative environment suitable for the cell proliferation and migration needed during wound healing. The use of dECM has an intrinsic bioactive capacity, thanks to its high similarity to the physiological ECM, which is a key factor in ensuring a correct wound healing. In fact, analysis of its composition shows the highly complex content of this biological scaffold, mainly comprised of type I collagen, but with significant presence of other ECM components such as elastin and glycosaminoglycans, among which a significant amount is represented by hyaluronic acid. To conclude, the results prove that this is a complex, rich and bioactive biomaterial, easily obtained from a food industry by-product, thereby also promoting the concept of the circular economy.

## Figures and Tables

**Table 1:** Forward and reverse primers used for RT-qPCR.

RNA	Forward Primer	Reverse Primer
CD80	5′-TGGTGCTGGCTGGTCTTTC-3′	5′-CTGTGCCACTTCTTTCACTTCC-3′
CD86	5′-ACATTCTCTTTGTGATGGCCTTC-3′	5′-TGCAGTCTCATTGAA ATAAGCTTGA-3′
CD163	5′-TCCACACGTCCAGAACAGTC-3′	5′-CCTTGGAAACAGAGACAGGC-3′
CD206	5′-CAGGTGTGGGCTCAGGTAGT-3′	5′-TGTGGTGAGCTGAAAGGTGA-3′
TNF-α	5′-CTGAACTTCGGGGTGATCG-3′	5′-GCTTGGTGGTTTGCTACGAC-3′
GADPH	5′-AACGTGTCAGTGGTGGACCTG-3′	5′-AGTGGGTGTCGCTGTTGAAGT-3′

**Table 2:** The order of the markers on the Human Angiogenesis Antibody Array.

A	B	C	D	E	F	G	H
POS	POS	NEG	NEG	Angiogenin	EGF	ENA-78	b FGF
POS	POS	NEG	NEG	Angiogenin	EGF	ENA-78	b FGF
GRO	IFN-γ	IGF-I	IL-6	IL-8	LEPTIN	MCP-1	PDGF-BB
GRO	IFN-γ	IGF-I	IL-6	IL-8	LEPTIN	MCP-1	PDGF-BB
PlGF	RANTES	TGF-β1	TIMP-1	TIMP-2	Thrombopoietin	VEGF	VEGF-D
PlGF	RANTES	TGF-β1	TIMP-1	TIMP-2	Thrombopoietin	VEGF	VEGF-D
BLANK	BLANK	BLANK	BLANK	BLANK	BLANK	NEG	POS
BLANK	BLANK	BLANK	BLANK	BLANK	BLANK	NEG	POS

**Table 3:** Functional score of the mice is shown for PBS 1X, collagen and 4 mg/mL dECM hydrogel conditions. The score was assessed visually at time points of 2, 7, 14, 21 and 28 days after treatment. Score values and meanings are also given, according to the functional scoring system reported by Brenes et al.

**Tarlov Score 0-6**							
**Score**	**Description**	**Sample**	**T2**	**T7**	**T14**	**T21**	**T28**
0	No movement	*PBS*	1	1	1	1	1
1	Barely perceptible movement, non-weight bearing	*COLLAGEN*	2	2	3	3	4
2	Frequent movement, non–weight bearing	*dECM*	2	2	3	4	5
3	Supports weight, partial weight bearing						
4	Walks with mild deficit						
5	Normal but slow walking						
6	Full and fast walking						
**Ischemia Score 0-5**							
**Score**	**Description**	**Sample**	**T2**	**T7**	**T14**	**T21**	**T28**
0	Autoamputation > half lower limb	*PBS*	3	3	3	3	3
1	Gangrenous tissue > half foot	*COLLAGEN*	5	5	5	5	5
2	Gangrenous tissue < half foot, with lower limb muscle necrosis	*dECM*	5	5	5	5	5
3	Gangrenous tissue < half foot, without lower limb muscle necrosis						
4	Pale foot or gait abnormalities						
5	Normal						
**Modified Ischemia Score 0-7**							
**Score**	**Description**	**Sample**	**T2**	**T7**	**T14**	**T21**	**T28**
0	Autoamputation of leg	*PBS*	3	3	3	3	3
1	Leg necrosis	*COLLAGEN*	7	7	7	7	7
2	Foot necrosis	*dECM*	7	7	7	7	7
3	Discoloration of >2 toes						
4	Discoloration of 1 toe						
5	Discoloration of >2 nails						
6	Discoloration of 1 nail						
7	No necrosis						

## Data Availability

Data will be made available on contacting the corresponding author.

## Supplementary Materials

The following supporting information can be downloaded at: https://www.mdpi.com/article/10.3390/biom12091222/s1, Videos regarding the progress of HLI models over the course of 28 days (Section 3.3.3), treated with either PBS (Video S1, PBS treated HLI model), collagen (Video S2, Collagen treated HLI model) or dECM (Video S3, dECM treated HLI model), are provided.
