# Supplementary figures and images for: Regenerative Potential of A Bovine ECM-Derived Hydrogel for Biomedical Applications
Source: Biomolecules. 2022 Sep 2;12(9):1222. doi: 10.3390/biom12091222 (PMC9496624; doi:10.3390/biom12091222)

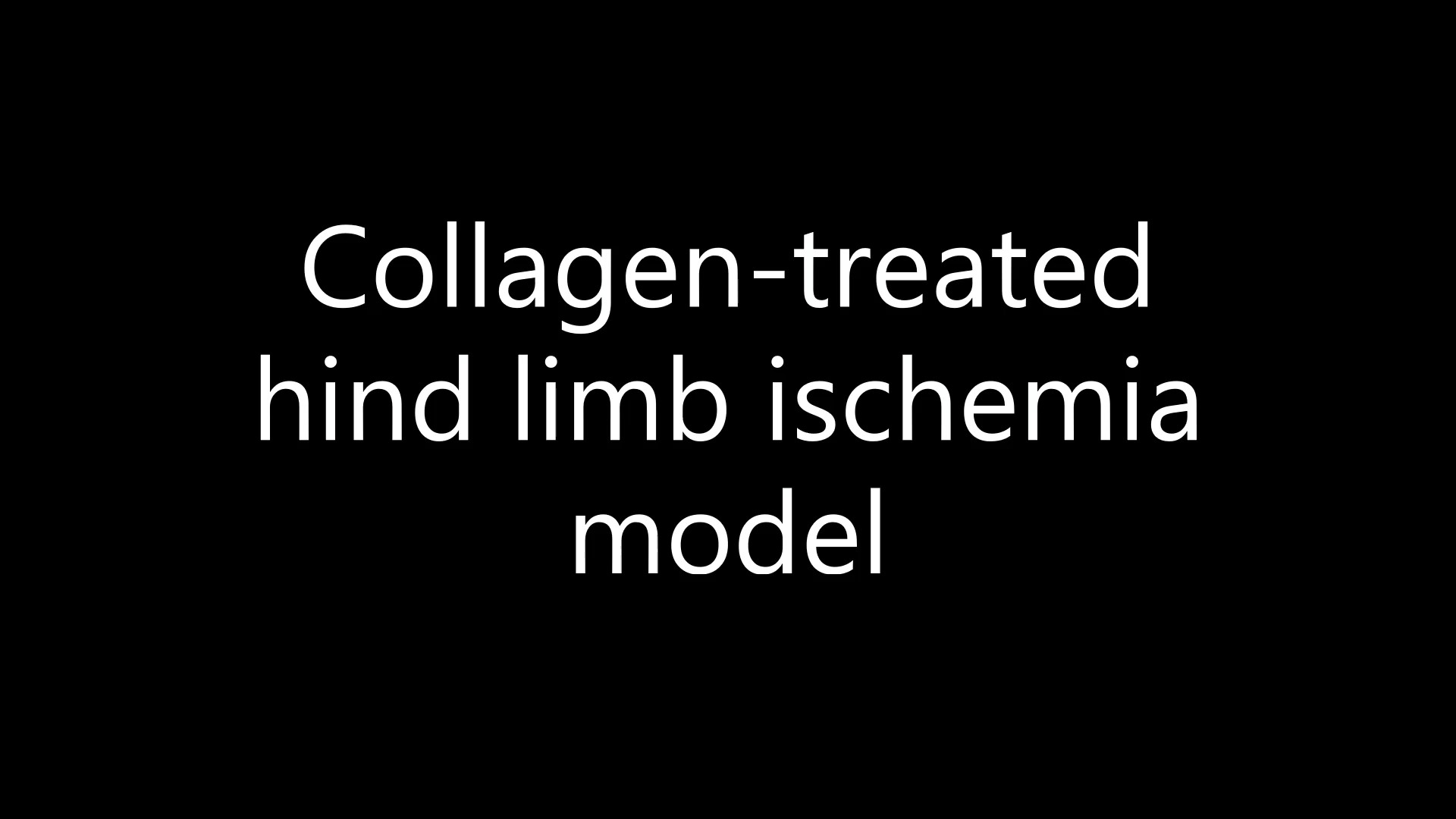

Supplement: Supplementary file 1 [file biomolecules-12-01222-s001.zip › Collagen treated HLI model DAY 1 to 28_Moment.jpg]

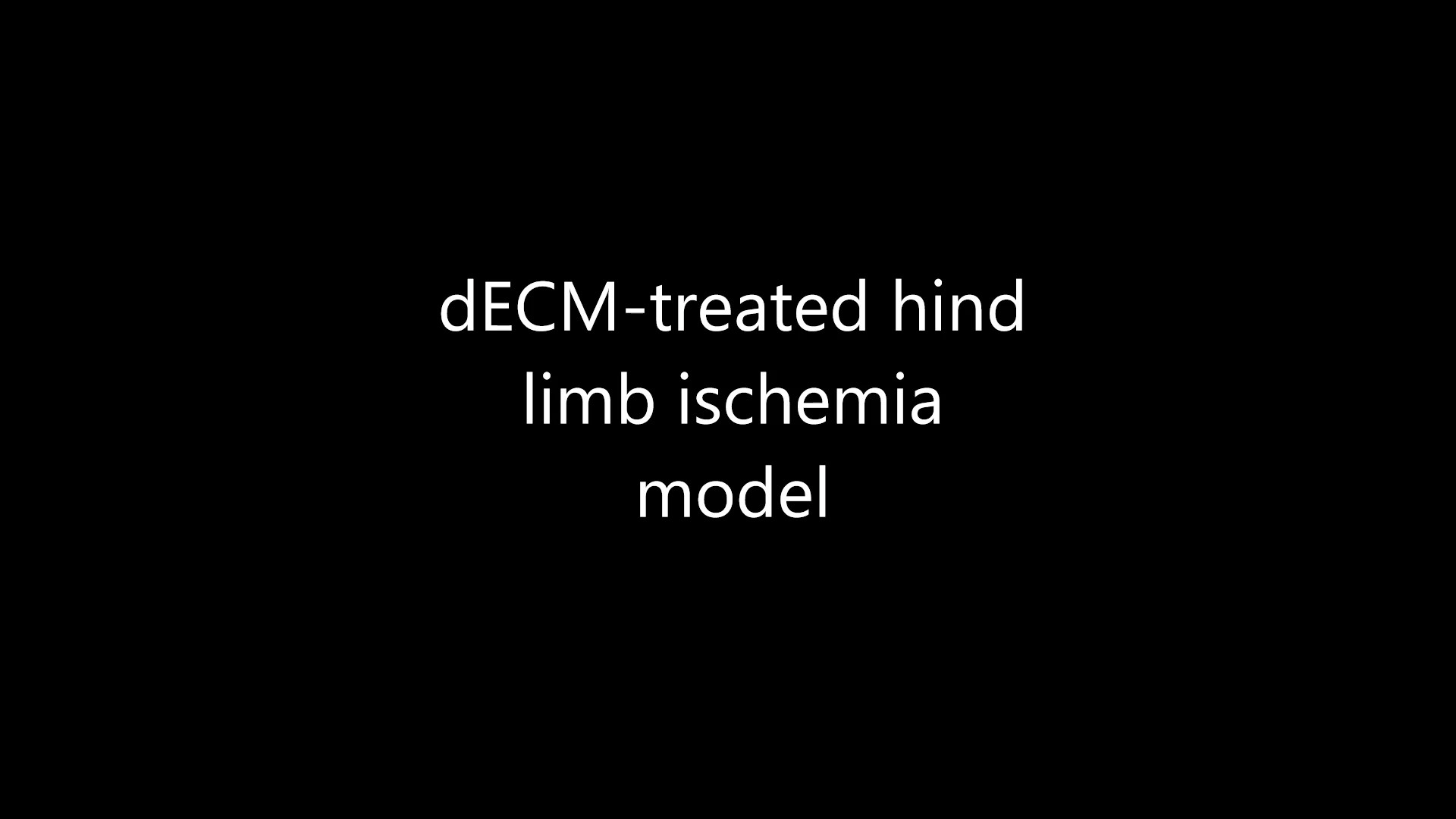

Supplement: Supplementary file 1 [file biomolecules-12-01222-s001.zip › dECM treated HLI model DAY 1 to 28_Moment.jpg]

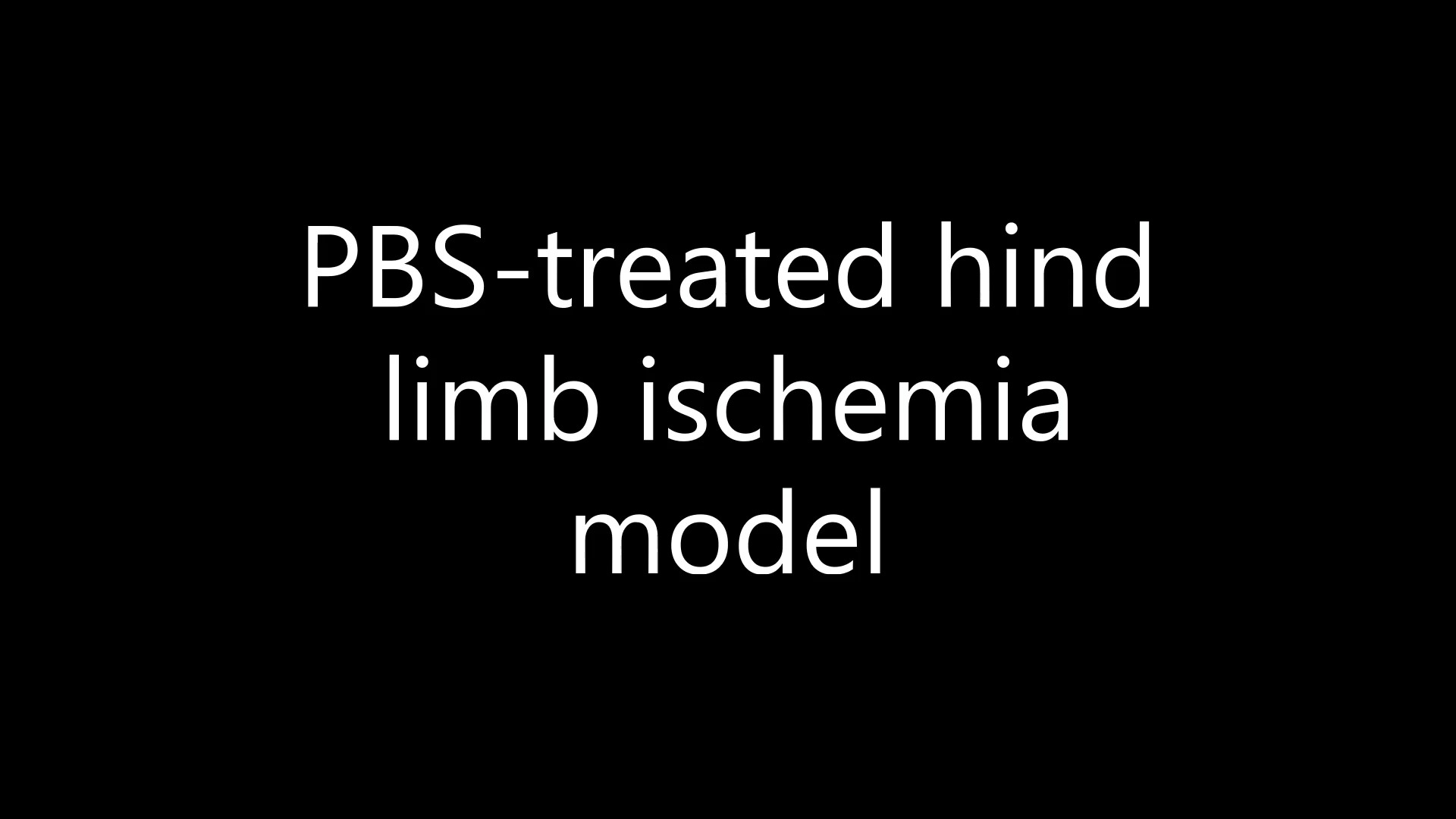

Supplement: Supplementary file 1 [file biomolecules-12-01222-s001.zip › PBS treated HLI model DAY 1 to 28_Moment.jpg]
